# Difference between 5A score and the HOPE score

**DOI:** 10.1186/s40560-022-00636-1

**Published:** 2022-09-09

**Authors:** Yohei Okada, Tasuku Matsuyama, Kei Hayashida, Shuhei Takauji, Jun Kanda, Shoji Yokobori

**Affiliations:** 1 Japan Association of Acute Medicine Heatstroke and Hypothermia Surveillance Committee, Tokyo, Japan; 2 Department of Preventive Services, Graduate School of Medicine, Kyoto University, ShogoinKawaramachi54, Kyoto, Sakyo 606-8507 Japan; 3 Department of Emergency and Critical Care Medicine, Japanese Red Cross Society Kyoto Daini Hospital, Kyoto, Japan; 4 Department of Emergency Medicine, Kyoto Prefectural University of Medicine, Kyoto, Japan; 5 Department of Emergency and Critical Care Medicine, Keio University School of Medicine, Tokyo, Japan; 6 Department of Emergency Medicine, North Shore University Hospital, Northwell Health System, Manhasset, NY USA; 7 Department of Emergency Medicine, Asahikawa Medical University Hospital, Asahikawa, Japan; 8 Department of Emergency Medicine, Teikyo University Hospital, Tokyo, Japan; 9 Department of Emergency and Critical Care Medicine, Nippon Medical School, Tokyo, Japan

## Abstract

Recently, a letter to the editor was published to comment on the 5A score which is the prediction model for accidental hypothermia patients comparing the HOPE score. In this letter, we responded to the comments to clarify the difference between the 5A score and the HOPE score.

Dear Editor,

We appreciate Pasquier et al. for their interest in the 5A score, a prediction model for in-hospital mortality among accidental hypothermia patients, derived by us, and for suggesting a discussion comparing the HOPE score in their letter [1–3]. The HOPE score derived by Pasquier et al. caught our attention since the article on the score was published, and we feel honoured to respond to their comments and clarify the role of the 5A score compared to that of the HOPE score [4, 5]

First, we agree with the conclusion of the letter that the target populations are different, and the 5A and the HOPE scores are complementary rather than exclusive [3]. The target population for the 5A score involves patients with hypothermia (body temperature ≤ 35 °C) in an emergency department including cardiac arrest patients. Further, in the J-point registry database, based on which the 5A score was developed, the study participants are mainly older individuals [the median and interquartile range (IQR) of age is 79 (67–87)], with disturbed activities of daily living in almost one-third of the patients, and hypothermia onset in indoor-settings in most cases [1, 6, 7]. They are representative patients of super-aging societies like Japan. The 5A score includes the albumin value as one of the variables, which is characterised as the assessment of frailty and undernutrition for this elderly population. Meanwhile, the HOPE score is focusing on cardiac arrest patients who underwent rewarming with extracorporeal membrane oxygenation (ECMO) [4]. The participants in the study conducted for the HOPE score are mainly young (median and IQR of age is 35 years [16–55]), and most of the cases seem to be associated with outdoor activities such as immersion or avalanche occurrence. Therefore, we believe that the target population and clinical roles are different for these scores. Accordingly, it is not incorrect that the 5A score is currently the first prediction model externally validated using a different dataset for accidental hypothermia patients in the emergency department belonging to the super-aging society.

Second, Pasquier et al. commented that the discrimination ability of the HOPE score was superior to that of the 5A score [3]. We appreciate the extremely high performance of this score, and have also recognised that it was cited by the guidelines [8]. However, as mentioned above, the target populations and clinical roles differ between the scores; therefore, it is not simple to compare the predictive performance between them because the clinical spectrum of the target population or outcomes are different.

Third, Pasquier et al. mentioned a validation study in their letter [3]. We agree with the authors’ statement that most of the prediction models are not evaluated in the validation study, and their replicability and applicability to other settings are unclear. We respect that they published an external validation study of the HOPE scores to confirm the reproductivity of predictive performance [5, 9]. However, in this validation study, because the data collection was based on a systematic review of the literature, we were concerned about whether the study participants were representative of the target population and applicability to other settings. For example, in the validation study of the HOPE score, information on the study participants was derived from five different settings (Japan, Denmark, Poland, France, and Switzerland) and different periods (the oldest and newest patient data were collected from years 1994 and 2018, respectively). Further, as discussed in our paper, there might be a risk of selection bias due to the indication of extracorporeal rewarming and publication bias of the relevant literature [2]. Ideally, we consider it desirable to perform a prospective external validation study focusing on a certain target population to confirm its applicability. On the other hand, the 5A score was externally validated in a validation study in which patient characteristics were similar to those in the original development study using prospectively collected nationwide data in Japan [2]. Accordingly, we believe that the results of the 5A score are generalisable to other settings, similar to those in Japan.

We reiterate that the 5A score is not an alternative to the HOPE score, and clinicians should employ them based on the clinical context. If cardiac arrest patients with hypothermia are considered candidates for extracorporeal rewarming, we believe that HOPE is also valuable for decision-making.

## Data Availability

Not applicable.
